# Expression of ABA Metabolism-Related Genes Suggests Similarities and Differences Between Seed Dormancy and Bud Dormancy of Peach (*Prunus persica*)

**DOI:** 10.3389/fpls.2015.01248

**Published:** 2016-01-11

**Authors:** Dongling Wang, Zhenzhen Gao, Peiyong Du, Wei Xiao, Qiuping Tan, Xiude Chen, Ling Li, Dongsheng Gao

**Affiliations:** ^1^State Key Laboratory of Crop Biology, Shandong Agricultural UniversityTaian, China; ^2^College of Horticulture Science and Engineering, Shandong Agricultural UniversityTaian, China; ^3^Shandong Collaborative Innovation Center for Fruit and Vegetable Production with High Quality and EfficiencyTaian, China

**Keywords:** seed dormancy, bud dormancy, abscisic acid, ABA metabolic genes, peach

## Abstract

Dormancy inhibits seed and bud growth of perennial plants until the environmental conditions are optimal for survival. Previous studies indicated that certain co-regulation pathways exist in seed and bud dormancy. In our study, we found that seed and bud dormancy are similar to some extent but show different reactions to chemical treatments that induce breaking of dormancy. Whether the abscisic acid (ABA) regulatory networks are similar in dormant peach seeds and buds is not well known; however, ABA is generally believed to play a critical role in seed and bud dormancy. In peach, some genes putatively involved in ABA synthesis and catabolism were identified and their expression patterns were studied to learn more about ABA homeostasis and the possible crosstalk between bud dormancy and seed dormancy mechanisms. The analysis demonstrated that two 9-cis-epoxycarotenoid dioxygenase-encoding genes seem to be key in regulating ABA biosynthesis to induce seed and bud dormancy. Three *CYP707As* play an overlapping role in controlling ABA inactivation, resulting in dormancy-release. In addition, Transcript analysis of ABA metabolism-related genes was much similar demonstrated that ABA pathways was similar in the regulation of vegetative and flower bud dormancy, whereas, expression patterns of ABA metabolism-related genes were different in seed dormancy showed that ABA pathway maybe different in regulating seed dormancy in peach.

## Introduction

Compared with traditional cultivation, protected cultivation of fruit trees is more economical and efficient, and it is an important way to promote farmers' income and rural economic development. Thus, protected cultivation of fruit trees has increased in China in recent years. The peach (*Prunus persica*) originated in China and has been cultivated for about 3000 years (Zheng et al., 2014). Peaches are important fruit trees in China, being cultivated in about 20,000 hectares. Generally, peach seedlings and fruit can be produced under protected cultivation all year. The production of peach fruit is influenced by blooming time; only after dormancy is broken, peach trees can proceed with the normal life cycle. Therefore, determining the mechanism of dormancy and using this knowledge to develop practical measures to break the seed and bud dormancy would be useful in the protected cultivation of peach trees.

Dormancy is defined as the inability to initiate growth from meristems under favorable conditions (Rohde and Bhalerao, 2007). Lang et al. (1987) distinguished bud dormancy into three types: paradormancy, inhibited by distal organs; endodormancy, inhibited by the dormant structure itself; and ecodormancy, inhibited by an unfavorable environment. Both seed and bud dormancy have been studied, and both types of dormant organs must undergo induction of dormancy, deep dormancy and dormancy breaking. Only after completing the whole process of dormancy can perennial plants proceed with the normal growth and development, otherwise, abnormal physiological phenomena might occur, such as dwarf seedlings, inconsistency of seed and bud germination and decreased fruit quality. Fu et al. (2014) proposed that there are certain similar molecular mechanisms between seed and bud dormancy; indeed, the hormonal regulations of bud and seed dormancy are similar (Cooke et al., 2012). Multiple physiological and transcriptomic studies have revealed the involvement of ABA in bud dormancy (Rohde et al., 2007; Shalom et al., 2014; Zhu et al., 2015); however, few genetic studies support this view. By contrast, the involvement of ABA in seed dormancy has been confirmed using Arabidopsis mutants and other plants mutants (Finkelstein, 2013). In general, ABA plays an important role in biotic stress and abiotic stress responses, controlling a wide range of essential physiological processes. Furthermore, bud and seed dormancy are regulated by ABA in perennial trees to cope with the more extreme environment conditions encountered during autumn and winter. The level of ABA is regulated by its biosynthesis and catabolism. Previous studies have revealed the ABA regulatory network in plants.

### ABA synthesis and catabolism

**Figure 1:**
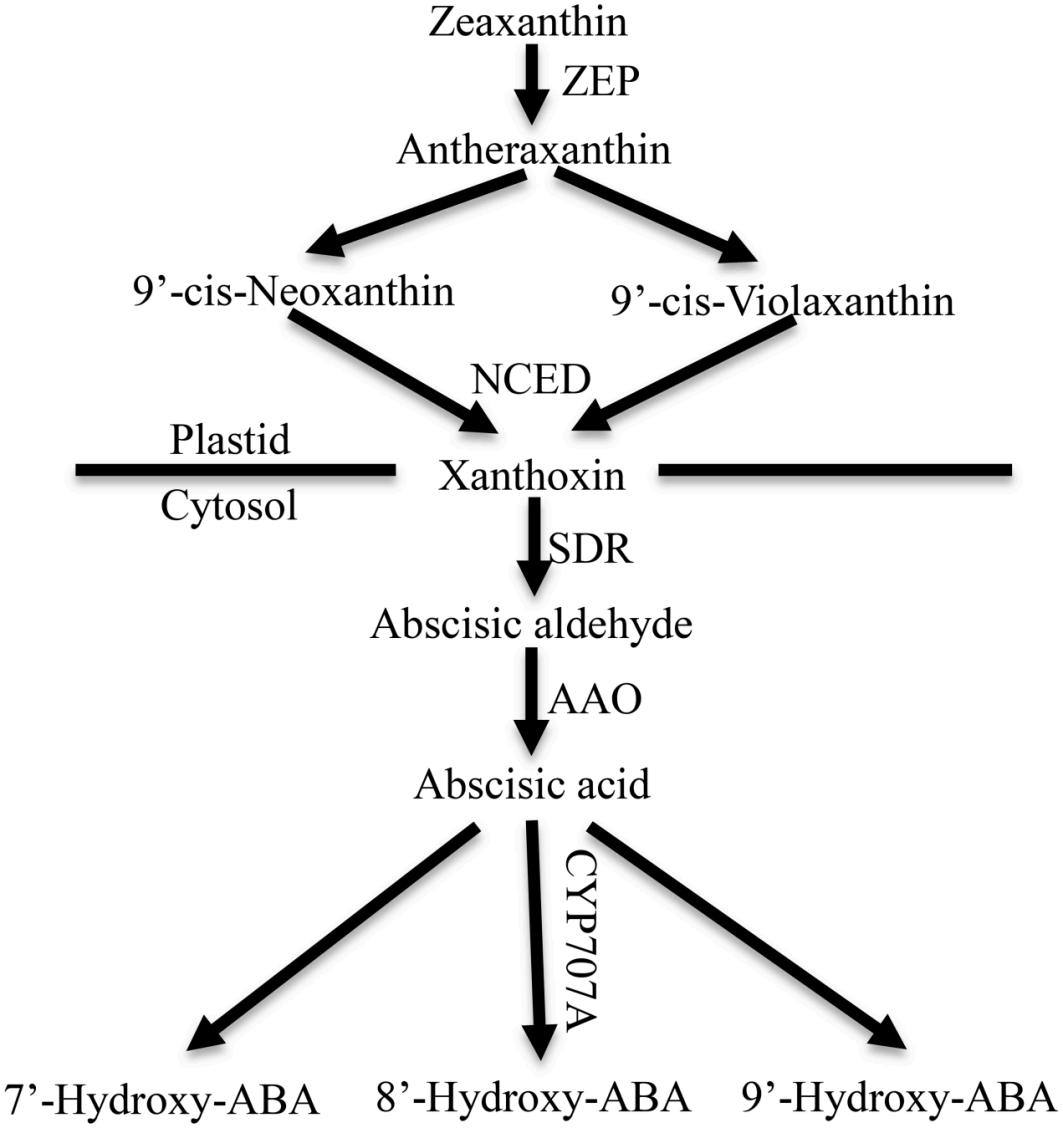
**ABA metabolic pathway in higher plants**. ZEP, zeaxanthin epoxidase; NCED, 9-cisepoxycarotenoid dioxygenase; SDR, short-chain dehydrogenase/reductase; AAO, aldehyde oxidase. CYP707A, abscisic acid-8′-hydroxylase (Leng et al., 2014).

ABA is synthesized *de novo* from a C40 carotenoid (Figure 1). The first step of ABA biosynthesis is epoxidation of zeaxanthin to all-trans-xanthophylls zeaxanthin and violaxanthin, catalyzed by zeaxanthin epoxidase (ZEP) in plastids (Koornneef et al., 1982; Marin et al., 1996). Violaxanthin is then converted to 9-cis-violaxanthin and 9-cis-neoxanthin, which are cleaved by 9-cis-epoxycarotenoid dioxygenase (NCED) to yield xanthoxin, the first C15 intermediate (Schwartz et al., 2003; Leng et al., 2014). Overexpression experiments in transgenic plants demonstrated that NCED is the key enzyme in limiting the rate of ABA synthesis (Thompson et al., 2000; Tung et al., 2008). Xanthoxin is transported to the cytosol, where it is converted to abscisic aldehyde by a short-chain alcohol dehydrogenase, which is encoded by *SDR1* (Cheng et al., 2002; González-Guzmán et al., 2002). Finally, abscisic aldehyde is oxidized to ABA by aldehyde oxidase 3 (encoded by *AAO3*) in the presence of a molybdenum cofactor (MOCO) (Seo et al., 2000; Bittner et al., 2001). In addition, ABA inactivation is the crucial mechanism that controls ABA level though oxidation or conjugation. ABA can be hydroxylated at the C-7′, C-8′ and C-9′ positions, and C-8′ is the major position for the hydroxylation reaction catalyzed by ABA 8′-hydroxylase (encoded by *CYP707A*) to yield 8′-hydroxy-ABA (8′-OH-ABA), which is unstable and enzymatically isomerizes to form phaseic acid (PA) (Kushiro et al., 2004; Saito et al., 2004).

### ABA and seed dormancy

In Arabidopsis, the regulation network controlling seed maturation seems to control the induction of seed dormancy (Rikiishi and Maekawa, 2014). ABA produced in the mature seeds regulates the induction of seed dormancy, and changes in the expressions of genes related to ABA synthesis and catabolism have been verified in Arabidopsis, bean, barley, and wheat (Chono et al., 2006; Millar et al., 2006; Yang and Zeevaart, 2006; Shu et al., 2013).

In higher plants, overexpression of certain genes related to ABA synthesis resulted in an increase of dry seed ABA content and delayed germination (Frey et al., 1999; Thompson et al., 2000; Qin and Zeevaart, 2002; Holdsworth et al., 2008). By contrast, their corresponding mutants showed ABA deficiency during various stages of plant and seed development, and exhibited greatly reduced seed dormancy: fresh seeds germinated at high frequency (Nambara et al., 1998; Lefebvre et al., 2006). Carotenoid cleavage by NCED plays an important role in ABA biosynthesis, and affects seed dormancy and germination. In Arabidopsis, nine NCED-related sequences have been identified, but only five gene members (*AtNCED2, AtNCED3, AtNCED5, AtNCED6, AtNCED9*) encode xanthoxin-producing enzymes, and *AtNCED5, AtNCED6*, and *AtNCED9* were reported to influence the development and dormancy of seeds (Tan et al., 2003; Lefebvre et al., 2006; Frey et al., 2012). By reporter gene analysis and *in situ* hybridization, *AtNCED6* was observed to be expressed in the endosperm, whereas, *AtNCED9* was expressed in both the endosperm and the embryo. ABA levels in *nced6* and *nced9* seeds were lower than in the wild-type, and *nced6nced*9 double mutants showed reduced seed dormancy (Lefebvre et al., 2006). Microarray data showed *AtNCED5* expression increased at late seed maturation stages, which was also confirmed by analysis of developing seeds of *pNCED5:GUS* transgenic plants (Frey et al., 2012). Overexpression of NCED in tobacco resulted in an increase in seed ABA content, and delayed the germination of imbibed seeds (Qin and Zeevaart, 2002).

ABA 8′-hydroxylase (encoded by *CYP707A*) is considered the key catabolic enzyme in ABA inactivation for controlling ABA level. In Arabidopsis, expression analysis found that *AtCYP707A1* was expressed in seeds at the mid-maturation stage and *AtCYP707A2* was expressed at late-maturation stage. In addition, ABA levels in dry seeds of *cyp707a1, cyp707a2*, and *cyp707a2 cyp707a3* double mutants were higher than in the wild-type, and the freshly harvest seeds of the *cyp707a* mutant showed a lower germination rate compared with the wild-type (Okamoto et al., 2006). In wheat, TM1833 (a double mutant of in *TaABA8*′*OH1-A* and *TaABA8*′*OH1-D*) showed lower *TaABA8*′*OH1* expression, higher ABA content during seed development and lower germination than “Tamaizumi” (a single mutant in *TaABA8*′*OH1-D*) (Chono et al., 2013). In barley, lower expression in transgenic ABA8′OH RNAi barley grains resulted in a higher ABA content and increased dormancy compared with non-transgenic barley grains (Gubler et al., 2008).

### ABA and bud dormancy

The role of ABA in controlling seed dormancy has been well established; however, its role in regulating bud dormancy is not well known. Until now, the progress of research on bud dormancy has been relatively slow because of its complex biological mechanisms.

The role of ABA in the regulation of bud endodormancy has been discussed in previous studies, and it is generally believed to be a key hormone in regulating bud dormancy (Cooke et al., 2012). In the autumn, the ABA content increases, resulting in cessation of shoot growth, promotion of apical buds set and induction of bud dormancy. In poplar, ABA levels reached their maximum after growth cessation in apical buds, accompanied by induction of genes related to ABA biosynthesis (*ABA1, NCED3, and ABA2*) (Arora et al., 2003; Ruttink et al., 2007). In the winter, buds remain dormant to inhibit bud growth to cope with the cold environment. During dormancy induction and maintenance of grape bud, ABA levels increased accompanied by increased expression of *VvNCED1*; however, the endogenous ABA levels decreased and ABA catabolites increased after buds achieved enough chilling accumulation, and *VvNCED1* was down-regulated and *VvCYP707A4* was up-regulated (Zheng et al., 2014). During peach bud dormancy release, *ppa005059m* and *ppa005020m* (highly similar to ABA 8′-hydroxylase genes *CYP707A*2 from Arabidopsis) were identified by suppression subtractive hybridization and microarray hybridization (Leida et al., 2010).

Using bud-breaking chemicals to study the mechanism of endogenous dormancy is an efficient way to demonstrate the relationship between ABA and bud dormancy. Although ABA is generally considered an important hormone for maintaining and releasing bud dormancy, there are conflicting results in some studies. In apple (Dutcher and Powell, 1972), and birch (Rinne et al., 1994), spraying ABA on buds resulted in delayed bud breaking; however, spraying ABA in grape buds had little effect on bud breaking (Hellman et al., 2006). Hydrogen cyanamide (HC) is a chemical that induces respiratory stress, leading to effective dormancy release. HC application reduced *VvNCED1* transcript levels and increased levels of *VvABA8*′*OH* homologs in grape, which was accompanied by reduced ABA content that resulted in a higher percentage of bud breaking than that in the control (Zheng et al., 2015).

### Gibberellins (GA) and ABA

In general, Gibberellin and ABA play an antagonistic role in regulating plant growth; Gibberellin is considered to promote plant growth, and ABA is considered to inhibit plant growth. In barely grains, addition of GA could promote germination during malting, and this was ascribed to increase of the enzymes relating to synthesis and secretion resulting in seed germination (Huang et al., 2016). In Arabidopsis, Cold stratification could increase GA level resulting in the release seed dormancy, and GA3ox1, a gene regulating GA biosynthesis, was induced by cold stratification (Nonogaki, 2014). Furthermore, previous studies had showed that the ratio of ABA and GA was relevant with seed dormancy and seed germination. In sweet cherry flower bud, ABA/GA increased with the process of endodormancy, reached maximum in the stage of deep dormancy, after that, it decreased with the broken of bud dormancy release (Duan et al., 2004).

Although ABA is confirmed to influence bud and seed dormancy, and the ABA pathway is quite clear, there have been few systematic studies of ABA metabolism in whole process of bud and seed dormancy, including their similarities and differences. In the current study, we monitored the expressions of genes related to ABA metabolism during the process of bud and seed dormancy in peach and demonstrated the relationship between seed dormancy and bud dormancy.

## Materials and methods

### Plant material

Peach trees (*Prunus persica* L. cv Zhong You Tao 4) were grown in an orchard located at the Shandong Institute of pomology in Taian, China under standard agricultural practices. As embryos appeared, 50 fruit were randomly collected from 30 distinct trees every 5 days and immediately dissected to remove the endocarp using special scissors. The seeds, seed coats and embryos were collected separately. Seed material was weighed and then frozen in liquid N_2_, and stored at −80°C until RNA extraction and quantitative real-time PCR (qRT-PCR) analysis.

Peach flower and vegetative buds (0.5 g) were sampled in late autumn (September) to mid-Spring (February) approximately every 15 days, from 9 a.m. to 11 a.m. The samples were immediately frozen in liquid N_2_ and stored at −80°C until RNA extraction and qRT-PCR analysis. Furthermore, at every time point, we randomly collected 30 1-year-old shoots and cultivated them in a vase containing 5% sucrose solution under a daily cycle of 25°C with artificial fluorescent light (200 μmol·m^−2^·s^−1^) for 16 h and 18°C in darkness for 8 h with constant 70% relative humidity to monitor the bud burst rate.

### Seed stratification at 4°C

Mature fruit (300) were collected and seeds with their endocarp were stratified at 4°C in wet gauze for 10 weeks to break dormancy. Stratified seeds (50 at each time point) were randomly brought out at a specified time interval and broken with a hammer to collect seeds, seed coats, and embryos. During the course of stratification, the seed germination rate was regularly examined at the optimum water supply and temperature (22°C) in the dark.

### Gibberellic acid (GA_3_) treatments

Mature seeds were soaked in 1 mM GA_3_ (Solarbio, Beijing, China) solution for 4 days. During this time, seeds were collected separately, washed three times with ultrapure water, weighed, frozen in liquid N_2_, and stored at −80°C. In addition, the control was soaked in ultrapure water. After treatment, the germination rate was tested under optimum water supply and temperature (22°C) in the dark.

### Analysis of the effect of hydrogen cyanamide (HC), high temperature (HT), gibberellic acid (GA_3_), and thidiazuron (TDZ) on bud break

On 22 October 2014, long 1-year-old branches were cut from Zhong You Tao 4 trees. Their basal parts were placed in 5% sucrose solution and incubated in a illumination incubator for a natural day length and under controlled conditions (conditions were the same as above). Subsequently, these branches were separated with five groups for treatments and every group contained 50 cuttings. 100 mL of 1 mM GA_3_(Solarbio, Beijing, China), 0.2 m MTDZ (KAYON, Shanghai, China) and 1% (v/v) HC (a solution containing 50% (w/v) HC, Sigma-Aldrich, St Louis, USA) with addition of 0.02% (v/v) Triton X-100 (Solarbio, Beijing, China) added as a surfactant were sprayed onto the branches, and then these treatments were transferred to the illumination incubator for an additional 30 d for bud-break monitoring. For HT treatment, cuttings were immersed in 50°C water for 1 h. In the control treatment, 0.02% (v/v) Triton X-100 was used as a spray.

### Identifying the genes related to ABA metabolism

Peach genomic sequencing was accomplished in 2013 (Verde et al., 2013); however, the functions of most of the genes are not clear. Few genes relating to ABA synthesis and catabolism have been identified. We used Endo's method (2014) to deduce the *NCEDs, CYP707As, AAOs, ZEP, SDR1* genes in the peach genome sequence (Endo et al., 2014). Firstly, a tblastp search was performed with one known amino acid sequence of each gene family from Arabidopsis in the peach genome at the Phytozome database (http://phytozome.jgi.doe.gov/pz/portal.html). This identified several candidate genes (*e* < 1e-10) of each family and their amino acid sequences were recorded. Secondly, a phylogenetic tree was constructed by Mega software version 6.0, using the neighbor-joining method and the protein sequences of candidate genes of each gene family. We then analyzed the phylogenetic tree and deleted those candidate genes whose genetic relationship was relatively distant. Thirdly, online SMART software at EMBL (http://smart.embl-heidelberg.de/) was used to determine whether each candidate gene's protein sequence had the typical conserved domains belonging to this family to further validate that these candidate genes belong to this family. Finally, the candidate gene protein sequences' similarities between Arabidopsis and peach were analyzed using DNAMAN software.

After these processes, *PpCYP707A1 (ppa005059m), PpCYP707A2 (ppa005020m), PpCYP707A3 (ppa005226m)*, and *PpCYP707A4 (ppa005234m)* were chosen as candidate genes of the CYP707A gene family. *PpNCED1 (ppa002804m), PpNCED2 (ppa002314m), PpNCED3 (ppa014647m)*, and *PpNCED4 (ppa006109m)* belonged to NCED gene family. *PpAAO1 (ppa000263m)* was the candidate gene of the AAO gene family. *ZEP* and *SDR1* are single genes belonging to the LOS gene family and SDR gene family, respectively. After analysis of these two gene families, the genetic relationship of *ppa002248m* and *ppa009814m* was quite close to *ZEP* and *ABA2*; therefore *ppa002248m* and *ppa009814m* were chosen as *PpZEP* and *PpSDR1* candidate peach genes.

### RNA extraction and qRT-PCR analyses

Total RNA was extracted from 0.2 g seeds, seed coats, embryos, and buds using a rapid plant RNA extraction kit (Aidlab, Beijing, China). The single-stranded cDNAs were synthesized from 1 μg of RNA using a cDNA synthesis kit according to the manufacturer's instructions (Takara Biotechnology, Dalian, China). qRT-PCR was performed with a gene specific primer pair and a β-*actin* primer pair as an internal control (Table 1). Reactions were performed on a CFX96 real-time PCR detection system with SYBR Premix Ex Taq (Takara). The thermo cycling parameters were as follows: 10 min at 95°C, followed by 40 cycles of 15 s at 95°C for denaturation and 1 min at 60°C for annealing and extension. The specificity of the PCR was assessed by the presence of a single peak in the dissociation curve after the amplification and by size estimation of the amplified product. The comparative cycle threshold (CT) method (2-ΔΔCT) method was used to quantify cDNAs with amplification efficiencies equivalent to that of the reference actin gene. Each experiment was repeated at least three times using the same cDNA source. For every reaction, the mean and SE values of relative transcript abundance were calculated. All the primers were designed by Beacon Designer 7 software. Results presented are the average of three independent biological replicates repeated three times.

**Table 1:** **Primers used to quantify the expression of the ABA metabolic genes and the reference genes**.

**Gene (Accession number)**	**Forward primer(5′–3′)**	**Reverse primer(5′–3′)**
*PpNCED1(ppa002804m)*	GACTTCGCTATCACAGAACGGTATG	CAGAGATGGAAGCAGAAGCAATCG
*PpNCED2(ppa002314m)*	GAAACCCAGAGACTCGTCCAAGA	AGCAGCCTGTCGTTGTGATAGA
*PpCYP707A1(ppa005059m)*	ACAGACCAACAGATTGCTGACAAC	CCTTCATCATCACCCTCCTCTTCTT
*PpCYP707A2(ppa005020m)*	TCGGCAACAATAGGGACTTTGAAATG	CCATCACTCTTCCTTCTCTTCGCTAT
*PpCYP707A3(ppa005226m)*	TCACCAAGGAGACTACCACAATAGC	CAAGGAAGCCAACATCAAAGGAGAAC
*PpZEP(ppa002248m)*	ACGAGGGCTTCCAGTTACAAGA	TGACCATATACCATCCGCTCCAA
*PpSDR1(ppa009814m)*	TGGCATTGGTGACTGGTGGA	GCACGGCTGACATCATCCTCTA
*PpAA01(ppa000263m)*	AGTTCTTGCGTACTCAGACTCGTT	ACTTCGTCAACAACAGGGTCATAC
*β-actin(ppa002399m)*	GTTATTCTTCATCGGCGTCTTCG	CTTCACCATTCCAGTTCCATTGTC

### Determination of endogenous (±) ABA

Two hundred milligrams of plant material was extracted in 1.8 ml cold phosphate-buffered saline (0.01 M, pH 9.2) (1:9, m/v), adjusted to pH 8.5. After centrifugation at 10,000 g (4°C, 10 min), the supernatant was filtered through 0.22 μm filter membrane. The efflux was collected for (±) ABA qualification, and ABA levels were quantified by Plant hormone abscisic acid (ABA) ELISA Kit (Bangyi Biotechnology, Shanghai, China), following the manufacturer's protocol. Each treatment consisted of three biological replications and each sample was measured with two technical replicates, and data from a typical experiment were presented.

### Statistical analysis

Statistical analysis was performed using SPSS for Windows version 19 (SPSS, Chicago, IL, USA). Categorical variables were expressed as frequencies, and percentages and continuous variables as the mean ± standard errors, as appropriate. The data were analyzed by ANOVA and, when appropriate, Duncan's test was used. A significance level of *p* < 0.05 was applied.

## Results

### Identification of peach ABA metabolism genes

ABA synthesis and catabolism genes had not been well annotated in the peach genome; therefore, we extracted annotated Arabidopsis genes from public database and searched for orthologs and paralogs from the peach database. Phylogenetic and protein domain analyses identified four *NCED*, four *CYP707A*, and one *AAO* gene family members. Table 2 lists the sizes of the full-length cDNAs and gDNAs, as well as the chromosomal locations of the candidate genes. Analysis of the genomic structure of the candidate genes related to ABA metabolism showed that the number of exons in the ABA-related genes in the available plant genomes is remarkably well conserved (Table S1). The degree of identity between the predicted amino acid sequences of each ABA-related catabolism gene and its respective orthologs in *Arabidopsis thaliana, Populus trichocarpa*, and *Oryza sativa* was calculated (Table S2). The amino acid sequences of the peach genes are very similar to members of the eudicots (e.g., *Arabidopsis and Populus*) and less similar to the monocot species (*Oryza*) (Table S2).

**Table 2:** **ABA metabolism-related genes in ***Prunus persica*****.

**Gene**	**Entry name**	**cDNA (bp)**	**gDNA (bp)**	**Annotation**	**Chromosome**
**ABA BIOSYNTHESIS**					
*PpZEP*	*ppa002248m*	2306	7078	Putative zeaxanthin epoxidase	VII
*PpSDR1*	*ppa009814m*	887	1889	Putative Xanthoxin dehydrogenase 2	II
*PpNCED1*	*ppa002804m*	2552	2552	Putative 9-cis-epoxycarotenoid dioxygenase 1	IV
*PpNCED2*	*ppa002314m*	2582	3060	Putative 9-cis-epoxycarotenoid dioxygenase 2	IV
*PpNCED3*	*ppa014647m*	1605	1605	Putative 9-cis-epoxycarotenoid dioxygenase 3	I
*PpNCED4*	*ppa006109m*	1847	2056	Putative 9-cis-epoxycarotenoid dioxygenase 4	I
*PpAAO1*	*ppa000263m*	4134	8989	Putative abscisic-aldehyde oxidase 1	VI
**ABA CATABOLISM**					
*PpCYP707A1*	*ppa005059m*	1786	2898	Putative (+)-abscisic acid 8′-hydroxylase 1	V
*PpCYP707A2*	*ppa005020m*	1867	5751	Putative (+)-abscisic acid 8′-hydroxylase 2	VI
*PpCYP707A3*	*ppa005226m*	1760	3036	Putative (+)-abscisic acid 8′-hydroxylase 3	VIII
*PpCYP707A4*	*ppa005234m*	1431	2706	Putative (+)-abscisic acid 8′-hydroxylase 4	I

### Phylogenic analysis of ABA metabolism genes

In Arabidopsis, *NCED* belongs to *CCD* gene family and there are nine *AtCCDs* (including five *NCEDs*). We identified four *NCED* genes in peach. *PpNCED1* and *PpNCED2* were located on chromosome 4 and *PpNCED3* and *PpNCED4* were located on chromosome 1 (Table 2). The amino acid sequence of Arabidopsis *AtNCED3* showed high similarity to *PpNCED1* and *PpNCED2* (61–67%), but relatively lower identities with *PpNCED3* and *PpNCED4* (27 and 32%). Phylogenetic analysis revealed that *PpNCED3* was quite similar to *AtNCED6*, and *PpNCED4* was similar to AtNCED4 (Figure S1).

The *CYP707A* gene family encodes ABA 8′- hydroxylases that control ABA catabolism, including four *CYP707A* genes in Arabidopsis (Saito et al., 2004), three in rice (Saika et al., 2007), two in peanut (Liu et al., 2014), three in bean (Yang and Zeevaart, 2006), three in potato (Suttle et al., 2012), five in maize (Vallabhaneni and Wurtzel, 2010), and 10 in soybean (Zheng et al., 2012). In our study, four genes were identified as candidate *CYP707A* genes in peach, among which *PpCYP707A1* and *PpCYP707A2* had been identified in a previous study (Leida et al., 2012). Multiple sequence alignments of the deduced amino acids of *PpCYP707A1* showed that it was quite similar to *AtCYP707A1* and *AtCYP707A2* (77 and 74% similarity, respectively) (Table S2 and Figure S1), and *PpCYP707A2* was similar to *AtCYP707A2* (66% similarity). *PpCYP707A3* and *PpCYP707A4* were similar to *AtCYP707A4* (70 and 67% similarity, respectively). Multiple sequence alignments of the deduced amino acids of the *CYP707A* gene family in peach showed 68% similarity. All of PpCYP707A gene family proteins contain the highly conserved cysteine residue (PFGNGTHSCPG), which is the putative heme iron ligand, and it is essential for catalysis.

Four *AAO* genes are present in Arabidopsis; however, we could only identify one ortholog in peach (Figure S1). Multiple sequence alignments of the deduced amino acid sequence of PpAAO1 showed 60, 61, 62, and 61% sequence identity with Arabidopsis *AtAAO1, AtAAO2, AtAAO3*, and *AtAAO4*, respectively.

*ABA2/SDR1* is a member of the *SDR* gene family in Arabidopsis; however, *SDR1* is thought to encode a unique protein that catalyzes the conversion of Xan (Xanthoxin) to ABAld (abscisic aldehyde) in Arabidopsis. We identified 10 *SDR*-like gene family members in peach, and constructed a phylogenetic tree using 20 amino acid sequences of the *SDR* family from peach and Arabidopsis. Phylogenetic analysis showed that *ppa009814m* was the most likely *SDR1*-like gene in peach (Figure S1). The deduced protein showed 71% sequence identity with Arabidopsis *AtABA2*/*AtSDR1*, and contained the N-terminal motif (Gly-X-X-X-Gly-X-Gly) and the Tyr-X-X-X-Lys motif that are characteristic of SDRs.

### Seasonal dormancy status of vegetative and flower buds of peach trees

To study how ABA-related genes changed during the whole process of dormancy of buds, the stages of bud dormancy should be precisely identified. In this study, 30 6-year-old peach trees in an orchard located at Shandong Institute of pomology were selected to study bud development. We used two methods to identify flower bud dormancy stage: measuring the increase in bud weight, including the bud water content, caused by initiation of meristem growth; the other was testing the percentage of bud breaking every 15 days from September to February in the next year caused by removal of environmental effects. We only use the latter method to identify vegetative bud development because peach vegetative buds are quite small.

**Figure 2:**
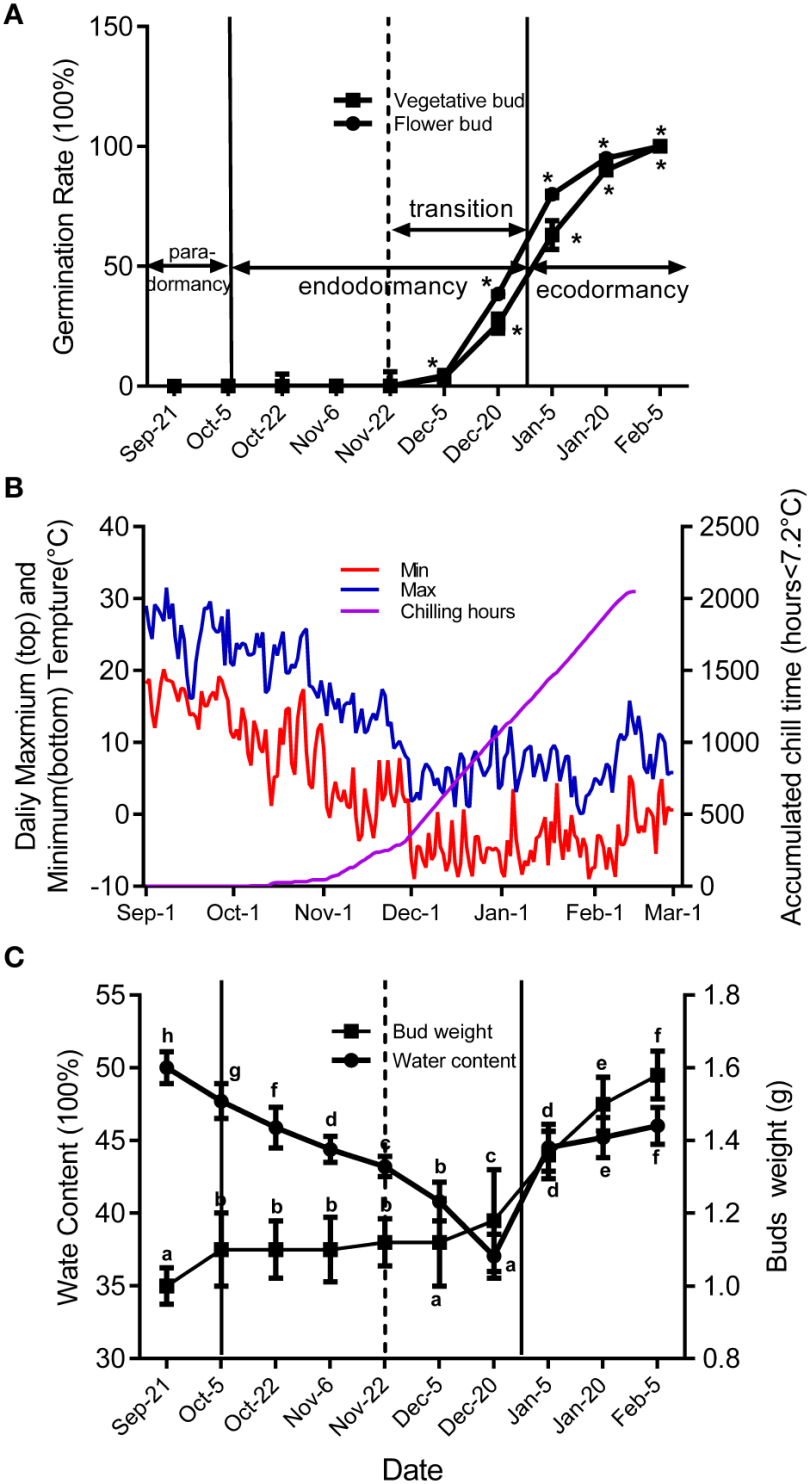
**Characterization of vegetative and flower bud condition and seasonal changes of weather conditions around orchard located at Shandong Institute of pomology**. Measurements are averages of three biological replicates with error bars representing the SEM. **(A)** Changes in dormancy status of the bud population throughout the dormancy cycle. Bud-break percentages at 25 d were presented to describe the seasonal changes in dormancy status of the bud population (^*^P < 0.05). **(B)** Daily maximum and minimum temperatures and cumulative chilling hours from September to the following February in 2014–2015. **(C)** Changes of average flower bud weight and water content from 21 September to 5 February. Different letters above bars indicate a significant difference among chilling periods according to ANOVA and Duncan's test (*P* < 0.05).

Figure 2 shows seasonal percentage of bud breaking in the 25-day after incubated in 5% sucrose solution. At the same time, the weather conditions and chilling accumulation (number of hours below 7.2°C) were recorded to understand the environmental effect on bud development (Figure 2B). From 27 September to 5 December, no flower buds or vegetative buds broke from 1-year-old shoots (accumulated chilling hours, 482 h), which we termed endodormancy, caused by inhibition by the dormant buds itself. After 22 November, the dormancy of the flower and vegetative buds started to be released. From 23 November to 15 January in the next year, the dormancy of flower buds and vegetative buds was fully released, with 80 and 63% of buds breaking, respectively. We termed this stage as the dormancy release period (transition stage), in which the weight of the flower bud increased (Figure 2C). After 15 January, the buds could burst, in theory; however, they did not burst because of the cold environment; this is known as ecodormancy, where bursting is inhibited by the disadvantageous environment. Figure 2A shows the buds sampled on 5 December that broke first; we considered this the key time point of bud dormancy release. In addition, flower bud dormancy release was earlier than vegetative bud dormancy release in peach trees, which could explain why peach blossoms emerge earlier than leaves under field conditions.

### Seasonal expression changes of ABA synthesis and catabolism genes in vegetative and flower bud dormancy

**Figure 3:**
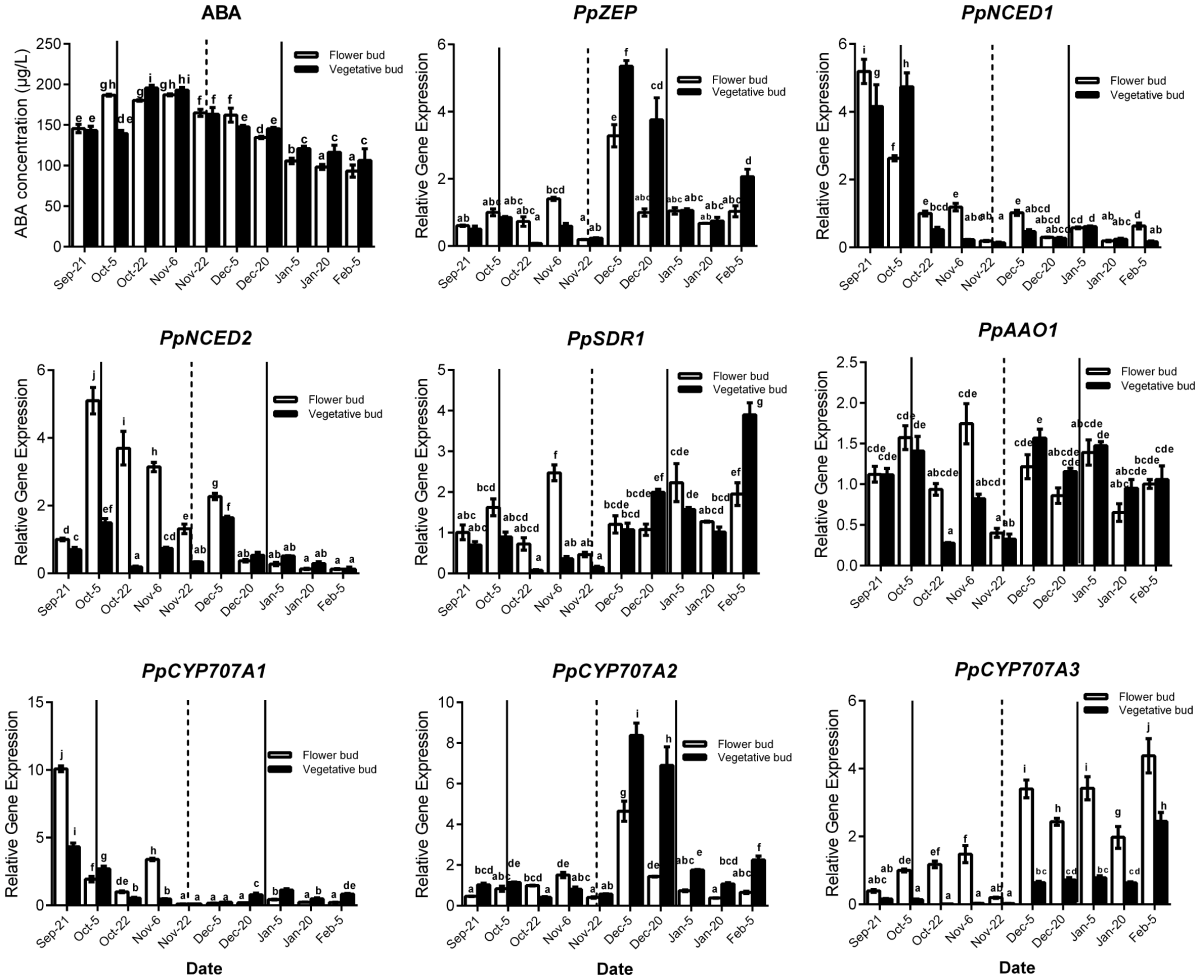
**Relative gene expression of eight ABA metabolic genes during the period of vegetative and flower bud development stage**. Each experiment was performed with three biological replicates with error bars representing the SEM. Different letters above bars indicate a significant difference among chilling periods according to ANOVA and Duncan's test (*P* < 0.05). The lines show buds development stage which has the same meaning with the lines in Figure 2.

Seasonal expression analysis of ABA synthesis and catabolism genes demonstrated that these genes were expressed and showed similar change trends between vegetative and lateral flower bud dormancy (Figure 3). Meanwhile ABA concentration in vegetative and lateral flower buds showed the similar trends, and both were high during the early part of the rest period and then gradually declined (transition stage), reaching the minimum concentration in the period of ecodormancy. *PpNCED1* and *PpCYP707A1* transcript levels showed the same change trend: both steadily decreased from September to February. *PpNCED2* was expressed at lower level on 21 September, and then increased dramatically to reach a peak on 4 October, after which it decreased gradually until February. The *PpSDR1* transcript level was relatively higher initially, but decreased on 22 October and remained at a relatively low expression level until 22 November, after which it increased to a high expression level. Both *PpZEP* and *PpCYP707A2* were expressed at low levels from 21 September to 22 October, and then increased suddenly on 5 December (the key time point of bud dormancy release), and then after 20 December, the expressions of *PpZEP* and *PpCYP707A2* returned to relatively lower expression levels. The transcript level of *PpAAO1* was always maintained at a high level during the period of vegetative and bud dormancy, and its expression showed no obvious change. The expression of *PpCYP707A3* increased gradually from September to February in the next year, with a dramatic peak on 5 December. In addition, we found that all of the genes were expressed at their lowest levels on 22 November [the first phase before the key phase of bud release (5 December)]. This experiment was performed 2 years and showed the same expression trends (data not shown).

### Expression patterns of ABA synthesis and catabolism genes during seed development

**Figure 4:**
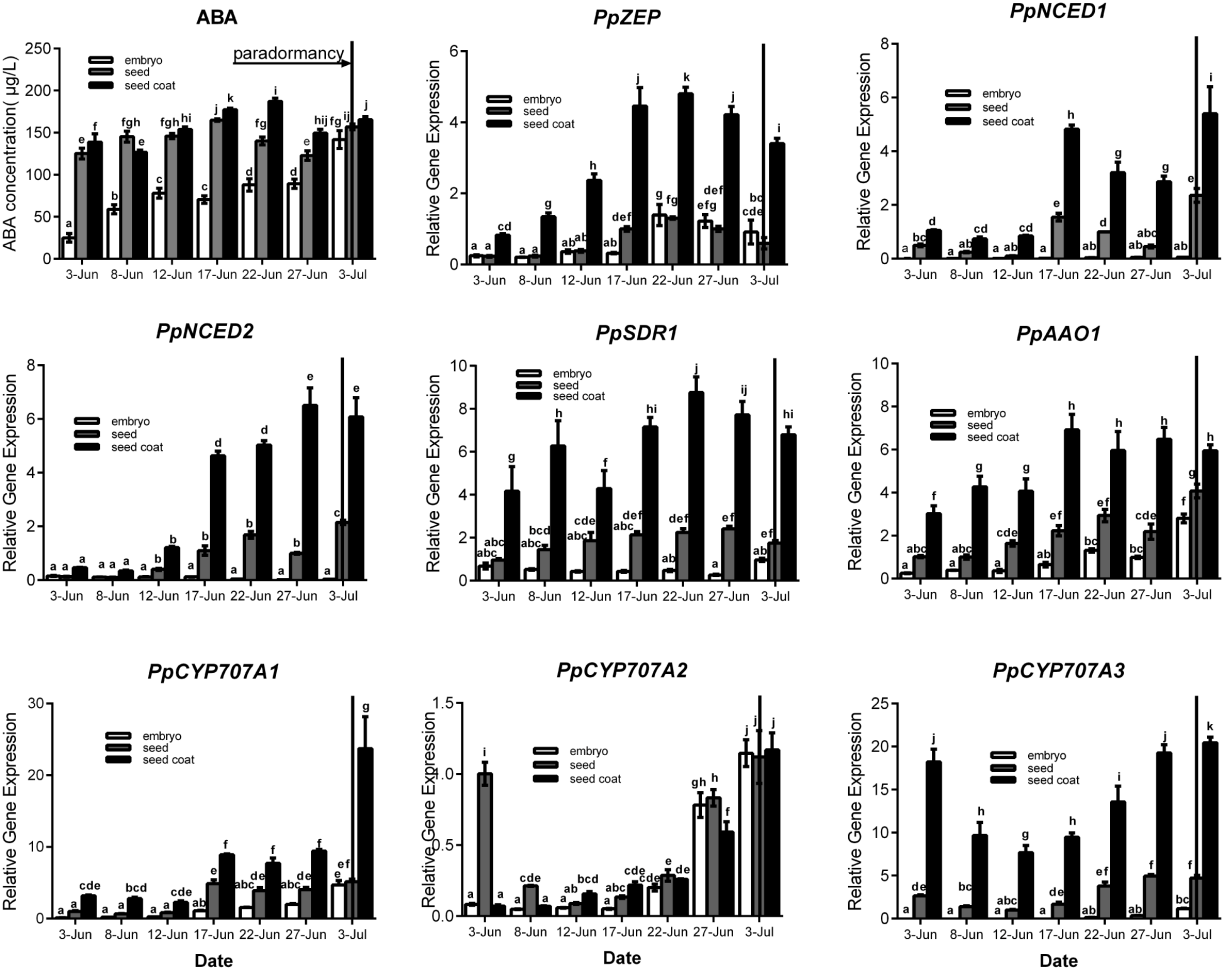
**Relative expression levels of eight ABA metabolic genes in seeds, seeds coat, and embryos during the period of seed development from 3 June to 3 July in 2015**. Seeds were sampled every 5 days. Expression levels were calculated relative to a peach β-actin as Figure 3, and data are presented as the means ± standard errors. Each experiment was performed three times. Different letters above bars indicate a significant difference among chilling periods according to ANOVA and Duncan's test (*P* < 0.05). In addition, the period of seeds development [paradormancy (induction of seed dormancy)] corresponds to buds paradormancy (induction of bud dormancy). The lines have the same meaning.

Seeds were sampled once embryos appeared (7 June), and seeds were separated into seed coat and embryo for analysis. For ABA qualification, data showed that ABA was gradually increased in seed coat, seed and embryo during seeds development, and ABA concentration in seed coat was higher than embryo (Figure 4). In the embryo, the expressions of *PpZEP, PpSDR1, PpAAO1, PpCYP707A1, PpCYP707A2*, and *PpCYP707A3* increased as the seeds developed (Figure 4). In seed coat, all these gene expression was higher than in seed and embryo. *PpNCED1, PpNCED2, PpCYP707A1*, and *PpCYP707A2* was expressed at a low level at the beginning, and then increased to a high transcript level at six-fold, 20-fold, 11-fold, 20-fold higher than the baseline level in 3 July, respectively. The transcript levels of *PpZEP, PpSDR1*, and *PpAAO1* gradually increased to a peak on 17 June, after which they decreased slightly. The expression of *PpCYP707A3* was high in the beginning, and then decreased until 12 June, after which it increased as the seeds developed (Figure 4). In addition, we observed that endosperm disappeared by the 12 June. Figure 4 demonstrates that all the genes were expressed at a relatively low level in early seed development, and then gradually increased to high expression levels, except *PpCYP707A2*. *PpCYP707A2* was expressed at a high level in the beginning, dramatically decreased to a low level, and then gradually increased to a peak at the end of seed development. In 3 June and 8 June, the endosperm was still present within seeds except embryo and seed coat; therefore, we measured these gene expressions during the process of endosperm abortive (data not shown). We found that *PpCYP707A1, PpCYP707A3, PpNCED1*, and *PpNCED2* were not expressed in late endosperm development in peach. *PpZEP, PpSDR1*, and *PpAAO1* expression increased from 3 to 8 June; however, *PpCYP707A2* showed the reverse trend, which was why *PpCYP707A2* transcript being at a higher level compared with the other genes in seeds from 3 to 8 June.

### Effect of stratification on seed germination and expression patterns of ABA metabolic genes during seed stratification

**Figure 5:**
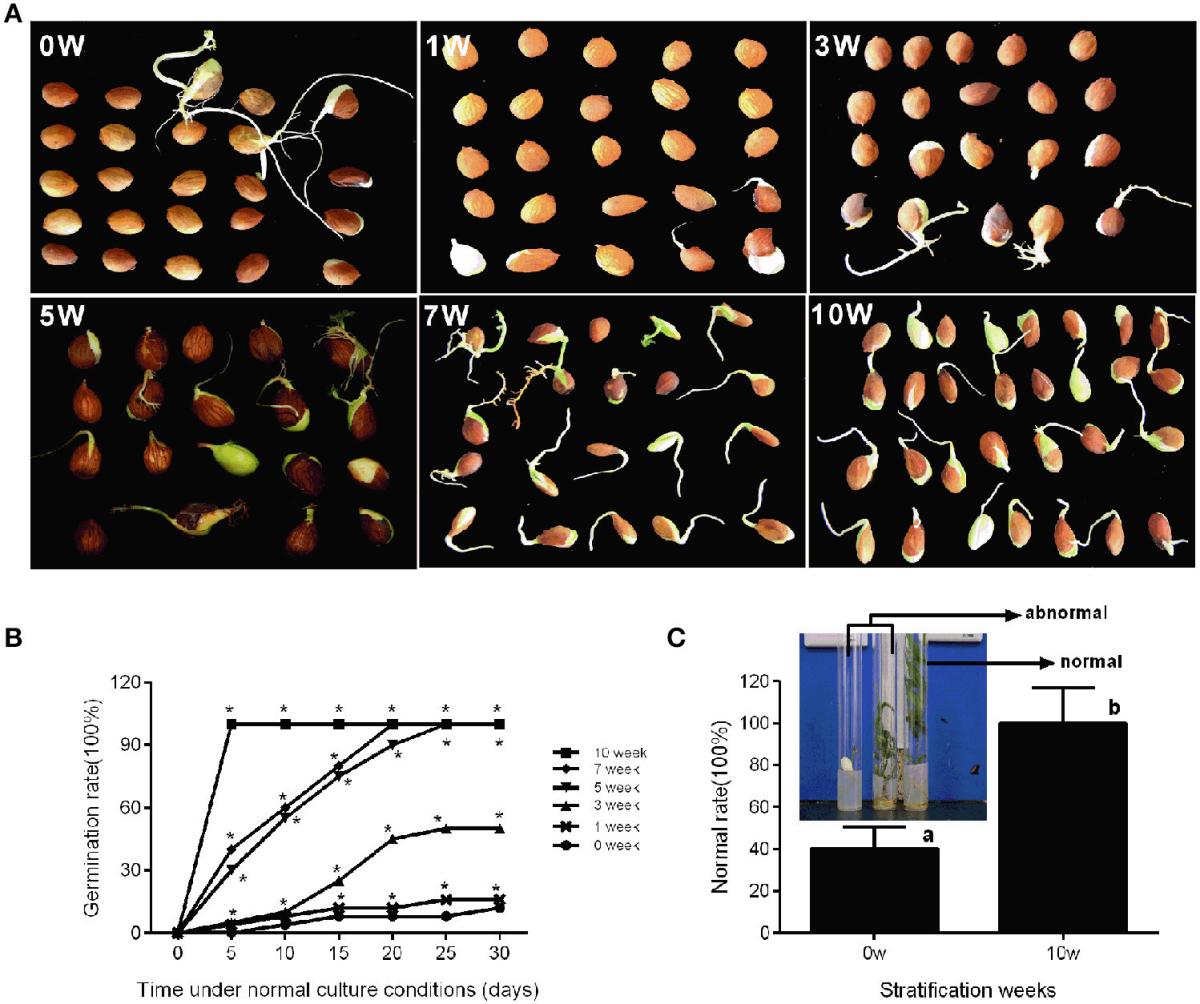
**Characterization of seed conditioning and germination after discrete periods of stratification**. The phenotype **(A)** and germination percentage of seed germination **(B)** were measured after discrete periods of stratification. **(C)** The normal rate of seedling after *in vitro* culture experiment of stratified embryo (10w) and non-stratified embryo (0w). The picture showed the normal seedlings and abnormal seedlings. Letters and asterisk above bars indicate a significant difference among chilling periods according to ANOVA and Duncan's test (^*^P < 0.05).

In production, insufficient chilling accumulation may be result in lower germination rate or disturbed emergence. In this study, a germination experiment was performed to characterize the response of seeds to different cold stratification periods. Seeds sampled from different stratification periods were placed in petri dish filled with wet gauze and cultured in the dark at 22°C. Figure 5B shows that the germination rate was low after 0, 1, and 3 weeks of stratification with 12, 16, and 50%, respectively. After 5 and 7 weeks of stratification, seed germination rate could reach 100%; however, the root length and germination time were not consistent (Figure 5A). After 10 weeks of stratification, seeds consistently germinated with 100% germination rate and root length was much the same on the 5th day (Figure 5A). In addition, we measured the difference between stratified seeds and non-stratified seeds through an *in vitro* culture experiment. After 10 weeks' stratification, the seedling formation rate reached 100%, whereas that of non-stratified seeds was much lower at 20% (Figure 5C). Stratification improved the seedling formation rate, shoot growth, the consistency of germination, and root length.

**Figure 6:**
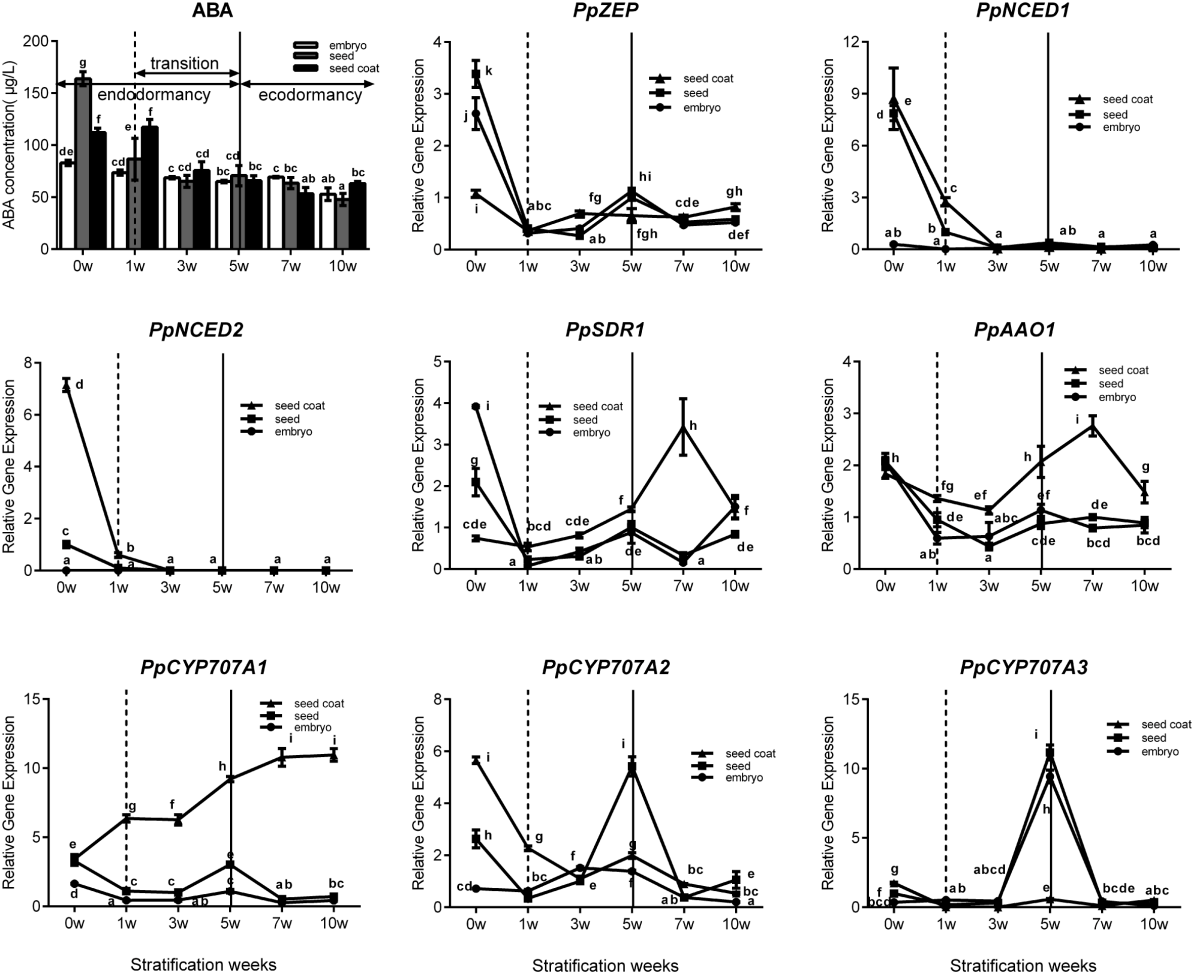
**Expression analysis of eight ABA metabolic genes in seeds, seeds coat, and embryos during the period of seed stratification**. Each experiment was performed with three biological replicates with error bars representing the SEM. Different letters above bars indicate a significant difference among chilling periods according to ANOVA and Duncan's test (*P* < 0.05). The lines have the same meaning.

Seed stratification resembles the bud dormancy release process, and also requires sufficient chilling accumulation, otherwise, some physiological abnormalities could occur during cultivation. In the period of seeds stratification, ABA concentration was gradually decreased in seed, embryo, and seed coat (Figure 6) which were the same with ABA changes in vegetative and lateral flower bud in the period of dormancy release (Figure 3). In addition, we also analyzed expression during seed stratification to assess whether ABA synthesis and catabolism genes performed similarly in stratification compared with bud dormancy. The transcript levels of *PpZEP, PpNCED1*, and *PpNCED2* were high at the start, and then decreased in seeds and seed coats during stratification (Figure 6). In seeds and embryos, *PpSDR1* and *PpAAO1* expression gradually decreased, but in seed coats their expression levels were relatively stable from 0 to 3 weeks, and then increased to a peak at 7 weeks (Figure 6)*. PpCYP707A* gene members showed different expression patterns during seed stratification (Figure 6). The expression level of *PpCYP707A1* showed the same trends during seed stratification in seeds and embryos. Initially, it decreased at 1 week of stratification, and then increased to a peak at 5 weeks (a key point in seed dormancy release), but in seed coat, the expression level of was steadily increased with seed stratification. The *PpCYP707A2* transcript level declined with seed stratification in seed coats; however, in embryos, its expression level gradually increased to a peak at 3 week. In seeds, the expression of *PpCYP707A2* was initially high, decreased in the first 3 weeks, and then dramatically increased to a peak at week 5. The expression of *PpCYP707A3* showed the same trends in seeds and embryos during seed stratification: the transcript was relatively stable in the first 3 weeks, and then dramatically increased to a peak at 5 weeks, with 55- and 90-fold higher expression compared with the baseline; however, in seed coats, the expression level was relatively stable.

### Effect of gibberellic acid on expression of central components of ABA synthesis and catabolism genes in seeds

**Figure 7:**
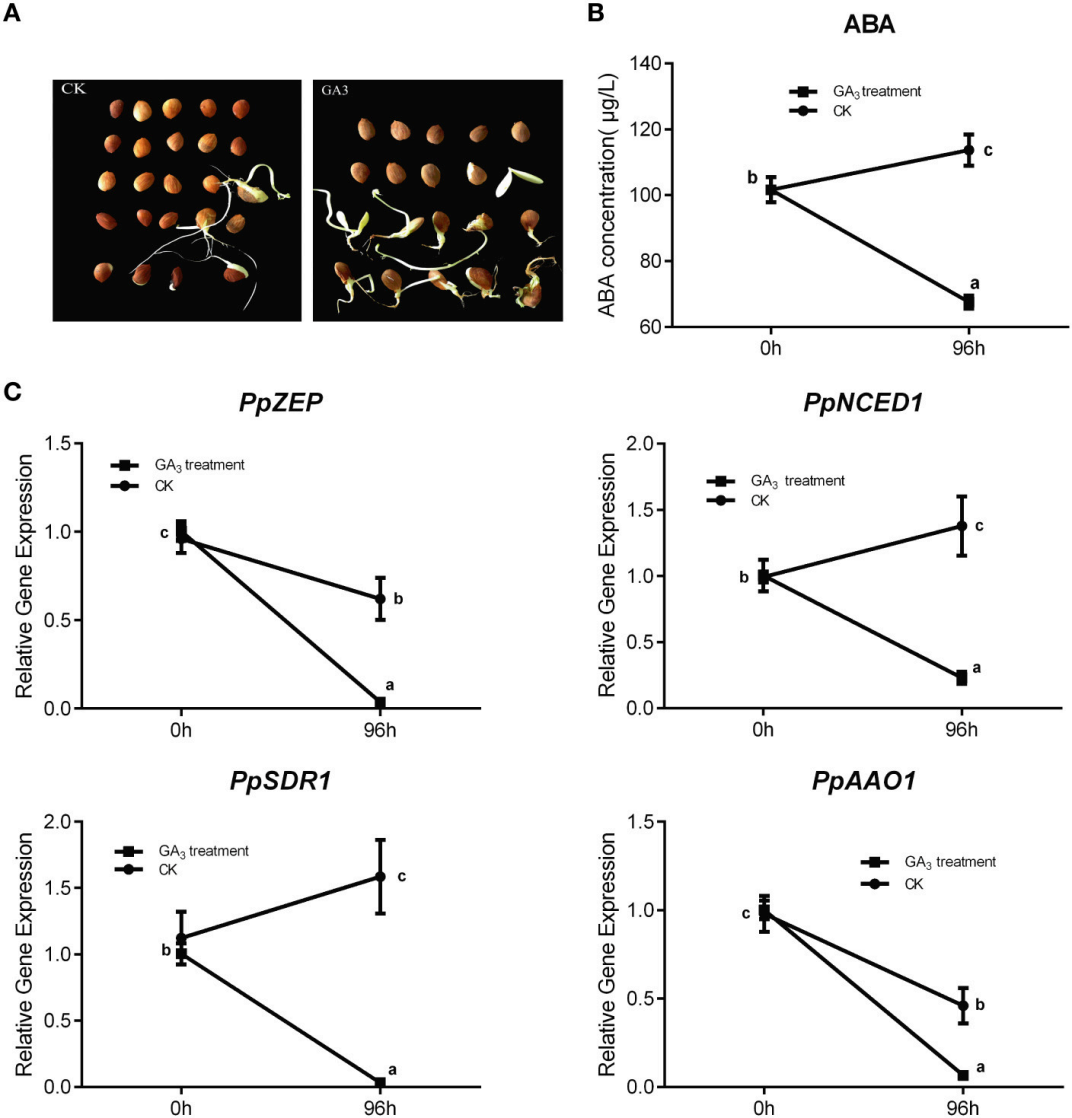
**The effect of GA_3_ on seed dormancy release and its role in relating ABA metabolism. (A)** Germination test was test after mature seeds treated with 1 mM GA_3_. **(B)** ABA changes after GA_3_ treatment in seed. **(C)** The expression analysis of ABA metabolic genes responding to GA_3_ treatment. Each experiment was performed with three biological replicates with error bars representing the SEM. Different letters above bars indicate a significant difference among chilling periods according to ANOVA and Duncan's test (*P* < 0.05).

In general, Gibberellin (GA_3_) and ABA play an antagonistic role in regulating seed dormancy and germination. In our study, mature peach seeds were soaked in 1 mM GA_3_ solution to study how Gibberellic acid affected the ABA metabolic network in regulating seed dormancy. We found that GA_3_ treatment resulted in seed dormancy release compared with the control (Figure 7A). Seeds treated with GA_3_ for 96 h showed 50% higher seed dormancy release compared with the control after 30 days of cultivation after treatment (Figure 7A). Even though GA_3_ treatment had an obvious role in breaking seed dormancy, it did not fully break seed dormancy, in contrast with 10 weeks' seed stratification (Figures 5A, 7A). Most seeds treated with GA_3_ showed the same phenotype as seeds with insufficient chilling accumulation. This illustrated that gibberellin could not fully break peach seed dormancy. In fruit production, a combination of Gibberellic acid treatment and stratification is usually used to shorten the peach seed dormancy period and fully break seed dormancy.

After GA_3_ treatment, data showed that ABA level was decreased comparing with control (Figure 7B), and qRT-PCR analysis showed that the expressions of *PpNCED1, PpZEP, PpSDR1*, and *PpAAO1* decreased compared with the control at 96 h (Figure 7C), whereas the transcript levels of all *PpCYP707As* showed no significant changes (data not shown).

### Effect of different measures on dormancy breaking of peach buds in the period of endodormancy

To test the effect of different dormancy breaking measures on peach bud dormancy, vegetative buds, and flower buds were treated with 1 m MGA_3_, high temperature (50°C), 1%HC, and 0.2 m MTDZ (thidiazuron). The data showed that different dormancy-breaking measures had different effects on flower bud and vegetative bud dormancy release (Table 3). For flower buds, HC treatment resulted in the earliest dormancy break and the highest bud burst rate at 25 days after treatment (90%), making it an effective way to break flower bud dormancy. 0.2 MTDZ treatment was less efficient (70% bud burst rate), and 50°C (15%) or 1 mM GA_3_ treatment (0%) were the least effective. For vegetative buds, 50°C treatment was the most effective way to break bud dormancy, with 70% of buds burst, and 1 mM GA_3_ and HC less effective (both 50% buds burst). 0.2 MTDZ had the lowest efficiency, with 2% buds burst at 25 days after treatment. In addition, 1 mM GA_3_and 1%HC also showed toxic effects on flower buds, which appeared as bark color changes, shoot tip drying and bud necrosis.

**Table 3:** **Bud burst percentage after different dormancy-breaking treatment**.

	**CK**	**GA^a^**	**HT^b^**	**TDZ^c^**	**HC^d^**
Flower buds	0	0	15%	70%	90%
Vegetative buds	0	50%	70%	2%	50%

a Vegetative and flower bud treated with 1 mM GA_3._

b *Vegetative and flower bud are treated with 50°C high temperature*.

c *Vegetative and flower bud treated with 0.2 mM TDZ*.

d *Vegetative and flower bud treated with 1%HC*.

## Discussion

### Relationship between vegetative bud and flower bud dormancy with ABA metabolic crosstalk

Dormancy defends woody plants against cold winters, protecting young meristems from freeze injury, and is indispensable in the vital process of buds life activity under natural conditions. In addition, buds in different phases, from dormancy to reactivation of growth, have different responses to environmental factors to adapting to local climatic conditions. In previous studies, plant hormones were confirmed to play key roles in regulating the bud dormancy cycle in woody plants (Rohde and Bhalerao, 2007; Cooke et al., 2012). ABA is the main hormone that responds to various environmental stresses, which affects the circadian clock, which in turn regulates plants sensitivity to ABA (Robertson et al., 2009; Cooke et al., 2012). Zheng et al. (2015) supported the assumption that the effect of ABA was dependent on the developmental stage of the buds. This would explained by the following three scenarios, or a combination of them: (i) the total ABA level increases because of the increase in endogenous ABA levels; (ii) the removal of the added exogenous ABA is caused by an increase of ABA degradation capacity; and (iii) ABA no longer controls primordial growth activity after developmental phase transition.

Peach bud dormancy is induced by the photoperiod, and the change in ABA level seems to be related to seasonal changes. In peach, continuous growth proceeded at 23/17°C day/night temperature in a 16-h photoperiod, but growth ceased and entry into dormancy began with a photoperiod of ≤ 13 h at the same temperature (Heide, 2008; Barros et al., 2012). In our previous studies, we considered that lateral buds went into dormancy induction stage in mid-September in Taian, China. At the same time, the day length was ~12.5 h, with average temperatures ranging from 28°C (maximum) to 15°C (minimum) and the transcript levels of *PpNCED1* and *PpNCED2* were high in vegetative and flower bud dormancy induction periods, resulting in increased ABA synthesis (Figures 2B, 3). In peach, ABA concentration increased with the development of bud dormancy and it gradually decreased with dormancy release, and reached the minimum concentration in ecodormancy (Figure 3). This result was the same as poplar dormancy, in which SD induced growth cessation and ABA levels increased dramatically after 3–4 weeks of short days in apical buds, in parallel with up-regulation of NCEDs and genes encoding enzymes catalyzing ABA biosynthesis (Rohde et al., 2002). We observed that *PpCYP707A1* was expressed at its highest level in the peach bud dormancy induction period (Figure 3); this might be relevant to water loss in the buds because CYP707A is involved in dehydration in many plants (Kushiro et al., 2004; Umezawa et al., 2006; Yang and Zeevaart, 2006). As the season progressed, the environmental temperature decreased and water loss continued in buds, resulting in deep dormancy of vegetative and flower buds in November; the increase in ABA level seemed to respond to the cold stress and waster loss. In deeply dormant potato tubers, the ABA content reached its highest level, which was also observed in grape and sweet cherry (Duan et al., 2004; Destefano-Beltrán et al., 2006; Zheng et al., 2015). When chilling accumulation reached about 300 h chilling requirements (CR) around 22 November), vegetative and flower buds burst under normal cultural environment in illumination incubator (Figure 2A). Thus, peach bud dormancy began to be released, and we considered that the period of bud dormancy release was from 22 November to 20 December, according to the bud germination test (Figure 2A). In this stage, the environmental temperature was lowest and living environment was poorest for buds, although they showed a potential to burst. In peach and grape, the ABA concentration declined during this period, which demonstrated that ABA inhibits bud burst (Ramina et al., 1994; Zheng et al., 2015). Our experimental data showed that the expressions of *PpCYP707A2* and *PpCYP707A3* were up-regulated during this stage to inactivate ABA, whereas *PpNCED2* was down-regulated, which would inhibit ABA synthesis. This was similar to grape dormancy release: treatment with HC resulted in bud dormancy release accompanied by a decrease in ABA levels in grape buds that paralleled the induction of *VvCYP707A4* expression and reduced *VvNCED1* expression (Zheng et al., 2015). Besides, *PpZEP* expression also dramatically increased in vegetative and flower bud in this period, seemingly in to response to cold weather: overexpression of the *ZEP* gene enhanced the ability to resist chilling stress in tomatoes (Wang et al., 2008; Figure 3). After dormancy release, peach vegetative and flower buds entered the stage of ecodormancy after 20 December. During this period, the buds swelled and showed increased metabolic activity and water content, and ABA was no longer an inhibitor of bud burst: the ABA concentration gradually decreased after bud dormancy release, possibly because *PpCYP707A3* was highly expressed to control the ABA content. At this stage, spraying ABA on peach vegetative and flower buds had no effect compared with the control, which were the same as the results with grape buds (data not shown).

In grape, ABA regulates bud dormancy and dormancy release by modifying ABA metabolism (Zheng et al., 2015). In our study, we found that *NCED* and *CYP707A* gene families are the key genes controlling ABA synthesis and catabolism. All the genes in our study showed similar expression patterns, which suggested that ABA crosstalk was comparable in vegetative and flower bud dormancy. The experiment was conducted in two different years (2013–2014 and 2014–2015) and all the genes related to ABA synthesis and catabolism were dramatically down-regulated on 22 November, before the buds entered the period of transition from endodormancy to ecodormancy (Figure 3); however, we cannot explain why this change occurred.

### High expression of ABA-related genes during seed development might induce seed dormancy

The central role of ABA in seed development and seed dormancy is well established and the key genes related to ABA synthesis and catabolism have been identified by studying plant mutants (Tan et al., 1997; Galpaz et al., 2008; Finkelstein, 2013). For most plants, ABA accumulates during seed development and declines during seed dormancy release (Kermode, 2005). To determine if the expressions of genes related to ABA metabolism correlated with seed ABA and dormancy levels, the expression patterns of certain ABA metabolic genes used to study bud dormancy were measured using qRT-PCR, and ABA concentration was measured by ELISA.

In Arabidopsis, the regulation network of the seed development program appears to control the induction of seed dormancy (Rikiishi and Maekawa, 2014). Generally, seed dormancy is classed into two major types: embryo dormancy and coat enhanced dormancy (Kermode, 2005). In peach seeds, the ABA concentration increased during the last developmental stage, and ABA accumulated in seed coats was higher compared with embryo (Figure 4). Our study found that all genes associated with ABA synthesis also increased during seed development, with the highest expression in the seed coat (Figure 4). Perhaps that was because a great quantity of ABA was synthesized in the seed coat to induce seed dormancy and prevent seed germination in late development. In Arabidopsis, *AtNCED6* and *AtNCED9* are required for ABA synthesis during seed development and show distinctive expression patterns. *AtNCED6* is expressed exclusively in the endosperm, whereas *AtNCED9* is expressed in testa and embryos (Lefebvre et al., 2006). In our study, *PpNCED1* and *PpNCED2* were found to be quite similar to *AtNCED9* through similarity analysis (Figure 2) and both of them were highly expressed in seed coats, but they were barely expressed in the embryo, endosperm, and embryo, which was the same as *AtNCED9*. In barley, the expression of *ABA2/SDR1*, a member of the *SDR* gene family, indicated its broader role in ABA synthesis. *HvSDR1, HvSDR2*, and *HvSDR4* were detected with high expression in embryos at various stages of seed maturation (Seiler et al., 2011); however, *SDR1* was not detected in the *Medicago truncatula* seed coat (Verdier et al., 2013), whereas we found that *PpSDR1* was expressed at low levels in the embryo and was highly expressed in the peach seed coat, which demonstrated that *SDR1* shows species specific expression patterns. In Arabidopsis, *AtAAO3* is the key gene catalyzing the final step of ABA biosynthesis in seed development, and ABA levels were low in the *aao3* mutant, which showed reduced dormancy, compared with the wild-type (Seo et al., 2004). In barley, five putative *AAO* gene family members were detected that participate in seed development, indicating their potential importance in seed ABA synthesis. Each of them plays different roles in different seed tissues (Seiler et al., 2011). Interestingly, we identified only one AAO gene in our study. Our data showed that *PpAAO1* expression increased in embryos, seeds and seed coats during late seed development (Figure 4) and increased during the period when the endosperm disappeared (data not shown). In addition to the genes related to ABA synthesis, ABA catabolic genes (*CYP707A*) were also induced during seed development to promote ABA inactivation (Figure 4). In Arabidopsis, *in situ* hybridization showed that *AtCYP707A1* mRNA could be detected in the vascular tissue in the embryo, whereas *AtCYP707A2* was found in the endosperm and vascular tissue in embryos during seed maturation (Okamoto et al., 2006). Similar to the *PvCYP707A* gene family in bean (Yang and Zeevaart, 2006), our data indicated that *PpCYP707As* also plays an overlapping role in peach seed development, Comparing the expression patterns of *CYP707As* in different seed tissues showed that *PpCYP707A2* is the main gene in the *CYP707A* gene family that participates in embryo development.

In late seed development, the genes relating to ABA synthesis are activated resulting in high ABA synthesis (Figure 4). Their high expression in the seed coat suggested that high levels of ABA in the seed coat induce embryo dormancy during late seed maturation. Thus, peach seed dormancy seems to be seed coat enhanced dormancy. Generally, some *CYP707A* gene family members showed increased expression that mirrored the ABA increase during tissue development (Chono et al., 2006; Millar et al., 2006; Chono et al., 2013). These observations agreed with our study, and we believe that the highly expression of *CYP707A* family members was to control of ABA content at normal level functions via inducing ABA inactivation and antagonizing high ABA synthesis.

### Peach seed dormancy termination is accompanied by expression changes in genes relating to ABA biosynthesis and catabolism

Stratification is an effective way to break seed dormancy in fruit trees. In a previous study, an *in vitro* culture experiment of peach seeds showed that seedlings would dwarf if chilling accumulation was insufficient (Leida et al., 2012). In general, seed dormancy release is accompanied by an obvious decrease in ABA content. The decline in ABA corresponded well with increasing germination capacity (Kermode, 2005). For example, the ABA content decreased in the embryo and megagametophyte during moist-chilling of Douglas fir seeds (Corbineau et al., 2002). In the dormancy termination of western white pine seeds, ABA and metabolite contents declined suddenly to relatively low levels in the seed coat after the pre-moist-chilling water soak, and in the embryo, the ABA content declined as ABA catabolism increased (Feurtado et al., 2004).

ABA catabolism is associated with dormancy release in some higher plants (Corbineau et al., 2002; Jacobsen et al., 2002; Feurtado et al., 2004); however, there are few reports on the molecular mechanism that regulates ABA metabolism to affect seed dormancy during moist-chilling. Previous studies on peach embryo dormancy showed that the ABA content dramatically declined after 1 week of stratification; however, the occurrence of seedling dwarfing did not improve even though ABA decreased to a quite a low level (Leida et al., 2012). In our study, we also found that ABA was decreased after at 1 week stratification, and then it gradually decreased and reach the minimum concentration after dormancy termination (Figure 6). All genes related to ABA synthesis were dramatically decreased after 1 week of stratification, and these changes corresponded to changes in ABA contents, especially for *PpNCED1* and *PpNCED2*, whose expression declined in seeds and seed coats during stratification (Figure 6). However, the expression pattern of other genes relating to ABA synthesis was quite different in seed coats, but they declined along with stratification as a whole in seeds and embryos (Figure 6). In Arabidopsis, the expression of *AtCYP707A* regulated seed ABA levels and dormancy depth, as deduced from analysis of the *cyp707a* mutant (Okamoto et al., 2006). Furthermore, seed dormancy increase under cold-maturation is related to *AtCYP707A2* down-regulation (Kendall et al., 2011). In our study, *PpCYP707As* showed different expression patterns in dormancy-release of peach seeds, which suggested that they have overlapping roles in ABA catabolism during bud dormancy release. *PpCYP707A1* was highly expressed in the seed coat, whereas *PpCYP707A2* and *PpCYP707A3* were mainly expressed in the embryo, suggested that *PpCYP707A1* and *PpCYP707A3* contribute to the inactivation of ABA in the seed coat and embryo, respectively. In addition, their expressions were dramatically increased at 5 weeks of stratification (a key time point in seed dormancy release), and the burst rate of seeds reached 100% at the same time, which suggests that they play a critical role in seed germination. Gibberellin and ABA were generally believed to have antagonistic effects on the regulation of dormancy breakage and germination. However, there was no report on the molecular mechanism of ABA catabolism in seeds treated with GA_3._ We found that only the genes relating to ABA synthesis were down-regulated in seeds treated with GA_3_ for 96 h accompanied with ABA decrease (Figure 7); thus GA_3_ might regulate seed dormancy by regulating ABA synthesis.

### Bud and seed dormancy are similar but different

Bud and seed dormancy are two different forms of dormancy that prevent pre-germination until environmental conditions become suitable for germination. Many studies have demonstrated similarities between seed dormancy and bud dormancy. For instance, in peach, expression analysis of genes identified by suppression subtractive hybridization during bud dormancy release showed similar expression patterns during embryo stratification (Leida et al., 2012). This demonstrated that there was a common regulation network in seed and bud dormancy. Moreover, expression of endoplasmic reticulum stress- and unfolded protein response-associated genes in bud endodormancy and seed stratification were similar in peach (Fu et al., 2014). ABA has been confirmed to control bud and seed dormancy as a stress hormone, and ABA accumulates during late seed development to induce seed dormancy in parallel with ABA accumulated in late autumn to induce bud dormancy. Furthermore, seed dormancy termination and bud dormancy release are associated with changes in ABA metabolism (Figures 3, 4). In the western white pine, dormancy termination of seeds is associated with changes in ABA metabolism (Feurtado et al., 2004), and grape bud dormancy is regulated by ABA through modification of ABA metabolism (Zheng et al., 2015). These observations supported the results of our study: peach seed and bud dormancy may be induced by two key genes contributing to ABA synthesis (*PpNCED1* and *PpNCED2*) and terminated by up-regulation of CYP707A gene family, resulting in ABA inactivation.

In our study, we found that vegetative buds and flower buds showed different bud burst rates after different chemical treatments, which suggested different regulation pathways between them (Table 3); however, ABA pathway crosstalk was suggested by the similar expression patterns in the two. In addition, HC could break the dormancy of both flower and vegetative buds, and GA_3_ could break vegetative and seed dormancy, which demonstrated there must be crossover point between these three different types of dormancy.

## Conclusions

Previous studies have demonstrated that ABA biosynthetic and catabolic genes play an important role in dormancy onset and release; however, there has been no systematic study on bud and seed dormancy that compared their differences. In our study, we found that *PpCYP707As* and *PpNCEDs* were the key genes in regulating peach seed and bud dormancy, which agreed with previous studies. The expression patterns of other genes indicated that their roles in regulating seed and bud dormancy are quite complex. During the induction of seed and bud dormancy, high expression of *PpNCED1* and *PpNCED2* might be associated with significant ABA synthesis, and the expressions of *PpCYP707A2* and *PpCYP707A3* suddenly increased during the period of dormancy-release maybe result in inactivation of ABA, which promotes seed germination and bud burst. Moreover, ABA crosstalk is similar between vegetative and flower bud dormancy, but different in seed dormancy, meanwhile the change trend of ABA concentration was much the same between seed dormancy and bud dormancy; this suggested that there might be another molecular mechanism contributing to ABA synthesis during bud dormancy. Even though we observed similarities and differences among the dormancy regulations of vegetative buds, flower buds, and seeds through studying ABA-related gene expressions, uncovering the integrated molecular network still requires further research.

## Author contributions

DW is the main author performed this work, and ZG contributed equally to this work. The others provided technical support and theoretical support to this work. LL and DG supervised the project.

### Conflict of interest statement

The authors declare that the research was conducted in the absence of any commercial or financial relationships that could be construed as a potential conflict of interest.
