# Supplementary material for: Expression of ABA Metabolism-Related Genes Suggests Similarities and Differences Between Seed Dormancy and Bud Dormancy of Peach (Prunus persica)
Source: Front Plant Sci. 2016 Jan 11;6:1248. doi: 10.3389/fpls.2015.01248 (PMC4707674; doi:10.3389/fpls.2015.01248)
Supplement: Supplementary file 1 [file Table1.DOCX]

**Expression of ABA metabolism-related genes suggests similarities and differences between seed dormancy and bud dormancy of peach (*Prunus persica*)**

# Dongling Wang^1,2^ ^†^, Zhenzhen Gao^1,2^ ^†^, Ling Li^1,2^, Peiyong Du^1,2^, Wei Xiao^1,2^, Qiuping Tan^1,2^, Xiude Chen^1,2^, Dongsheng Gao^1,2^*

^1^ State Key Laboratory of Crop Biology, Shandong Agricultural University, Taian, China

^2^ College of Horticulture Science and Engineering, Shandong Agricultural University, Taian, China

**^†^** These authors contributed equally to this work.

* **Correspondence:** Dongsheng Gao, College of Horticulture Science and Engineering, Shandong Agricultural University, Tai’an, Shandong, China

[dsgao@sdau.edu.cn](mailto:dsgao@sdau.edu.cn)

**Supplementary Table S1. The ABA metabolic genes: Gene families and the conservation of exon number**

|  | | | *Prunus*  *Persica*  Accession^1^ | *Arabidopsis*  *Thaliana*  Accession^1^ | *Populus trichocarpa*  Accession^1^ | *Oryza*  *Sativa*  Accession^1^ | Average^1^ |
| --- | --- | --- | --- | --- | --- | --- | --- |
|  |  |  | Exon (intron)^2^ | Exon (intron) | Exon (intron) | Exon (intron) | Exon number |
| Gene | Gene family^3^ | Sub family^4^ |  | | | |  |
| *PpZEP* | [HOM03D002699](http://bioinformatics.psb.ugent.be/plaza/versions/plaza_v3_dicots/gene_families/view/HOM03D002699) | [ORTHO03D004548](http://bioinformatics.psb.ugent.be/plaza/versions/plaza_v3_dicots/gene_families/view/ORTHO03D004548) | ppa002248m  16（16） | AT5G67030  16（16） | PT07G04430  17（16） | OS04G37619  16（15） | 16 |
| *PpABA2* | [HOM03D000124](http://bioinformatics.psb.ugent.be/plaza/versions/plaza_v3_dicots/gene_families/view/HOM03D000124) | [ORTHO03D008066](http://bioinformatics.psb.ugent.be/plaza/versions/plaza_v3_dicots/gene_families/view/ORTHO03D008066) | ppa009814m  2（1） | AT1G52340  2（1） | PT01G02430  2（1） | OS03G59610  2（1） | 2 |
| *PpNCED1* | [HOM03D000313](http://bioinformatics.psb.ugent.be/plaza/versions/plaza_v3_dicots/gene_families/view/HOM03D000313) | [ORTHO03D000541](http://bioinformatics.psb.ugent.be/plaza/versions/plaza_v3_dicots/gene_families/view/ORTHO03D000541) | ppa002804m  1（0） | AT3G14440  1（0） | PT01G39380  1（0） | OS03G44380  1（0） | 1 |
| *PpNCED2* | [HOM03D000313](http://bioinformatics.psb.ugent.be/plaza/versions/plaza_v3_dicots/gene_families/view/HOM03D000313) | [ORTHO03D000541](http://bioinformatics.psb.ugent.be/plaza/versions/plaza_v3_dicots/gene_families/view/ORTHO03D000541) | ppa002314m  2（1） | AT1G30100  1（0） | PT11G08410  1（0） | OS03G44380  1（0） | 1.25±0.5 |
| *PpNCED3* | [HOM03D000313](http://bioinformatics.psb.ugent.be/plaza/versions/plaza_v3_dicots/gene_families/view/HOM03D000313) | [ORTHO03D011932](http://bioinformatics.psb.ugent.be/plaza/versions/plaza_v3_dicots/gene_families/view/ORTHO03D011932) | ppa014647m  1（0） | AT3G24220  1（0） | PT03G17630  1（0） | OS03G44380  1（0） | 1 |
| *PpNCED4* | [HOM03D000313](http://bioinformatics.psb.ugent.be/plaza/versions/plaza_v3_dicots/gene_families/view/HOM03D000313) | [ORTHO03D001330](http://bioinformatics.psb.ugent.be/plaza/versions/plaza_v3_dicots/gene_families/view/ORTHO03D001330) | ppa006109m  2（1） | AT4G19170  1（0） | PT19G09340  1（0） | NA | 1.3±0.5 |
| *PpAAO1* | [HOM03D000693](http://bioinformatics.psb.ugent.be/plaza/versions/plaza_v3_dicots/gene_families/view/HOM03D000693) | [ORTHO03D000469](http://bioinformatics.psb.ugent.be/plaza/versions/plaza_v3_dicots/gene_families/view/ORTHO03D000469) | ppa000263m  10（9） | AT2G27150  10（9） | PT09G15380  10（9） | OS07G18120  10（9） | 10 |
| *PpCYP707A1* | [HOM03D000064](http://bioinformatics.psb.ugent.be/plaza/versions/plaza_v3_dicots/gene_families/view/HOM03D000064) | [ORTHO03D001028](http://bioinformatics.psb.ugent.be/plaza/versions/plaza_v3_dicots/gene_families/view/ORTHO03D001028) | ppa005059m  7（6） | AT4G19230  7（6） | PT04G23540  6（5） | OS02G47470  5（4） | 6.25±1 |
| *PpCYP707A2* | [HOM03D000064](http://bioinformatics.psb.ugent.be/plaza/versions/plaza_v3_dicots/gene_families/view/HOM03D000064) | [ORTHO03D000531](http://bioinformatics.psb.ugent.be/plaza/versions/plaza_v3_dicots/gene_families/view/ORTHO03D000531) | ppa005020m  8（7） | AT2G29090  8（7） | PT09G03390  7（6） | OS02G47470  5（4） | 7±1.4 |
| *PpCYP707A3* | [HOM03D000064](http://bioinformatics.psb.ugent.be/plaza/versions/plaza_v3_dicots/gene_families/view/HOM03D000064) | ORTHO03D00053 | ppa005226m  9（8） | AT3G19270  9（8） | PT04G14090  9（8） | OS09G28390  8（7） | 8.75±0.5 |
| *PpCYP707A4* | [HOM03D000064](http://bioinformatics.psb.ugent.be/plaza/versions/plaza_v3_dicots/gene_families/view/HOM03D000064) | [ORTHO03D000531](http://bioinformatics.psb.ugent.be/plaza/versions/plaza_v3_dicots/gene_families/view/ORTHO03D000531) | ppa005234m  9（8） | AT3G19270  9（8） | PT14G02910  9（8） | OS09G28390  8（7） | 8.5±0.6 |

^1^Sequence data from Phytozome 10.3: http://phytozome.jgi.doe.gov/pz/portal.html.

^2^Accession number from Plaza 3.0: (PLAZA v3: http://bioinformatics.psb.ugent.be/plaza/).

^3^ The gene family that the homologues are group into (PLAZA v3: http://bioinformatics.psb.ugent.be/plaza/).

^4^The sub-family that the orthologues group into (PLAZA v3: http://bioinformatics.psb.ugent.be/plaza/).
