# Supplementary material for: Expression of ABA Metabolism-Related Genes Suggests Similarities and Differences Between Seed Dormancy and Bud Dormancy of Peach (Prunus persica)
Source: Front Plant Sci. 2016 Jan 11;6:1248. doi: 10.3389/fpls.2015.01248 (PMC4707674; doi:10.3389/fpls.2015.01248)
Supplement: Supplementary file 2 [file Table2.DOCX]

**Expression of ABA metabolism-related genes suggests similarities and differences between seed dormancy and bud dormancy of peach (*Prunus persica*)**

# Dongling Wang^1,2^ ^†^, Zhenzhen Gao^1,2^ ^†^, Ling Li^1,2^, Peiyong Du^1,2^, Wei Xiao^1,2^, Qiuping Tan^1,2^, Xiude Chen^1,2^, Dongsheng Gao^1,2^*

^1^ State Key Laboratory of Crop Biology, Shandong Agricultural University, Taian, China

^2^ College of Horticulture Science and Engineering, Shandong Agricultural University, Taian, China

**^†^** These authors contributed equally to this work.

* **Correspondence:** Dongsheng Gao, College of Horticulture Science and Engineering, Shandong Agricultural University, Tai’an, Shandong, China

[dsgao@sdau.edu.cn](mailto:dsgao@sdau.edu.cn)

**Supplementary Table S2. Percentage identity of the predicted protein sequences of the ABA metabolic genes**

**(expressed relative to the *Prunus Persica* orthologue)**

|  | Accession number  Percentage identity relative to Prunus persica orthologue | | | |
| --- | --- | --- | --- | --- |
| Gene | *Prunus persica* | *Arabidopsis thaliana* | *Populus trichocarpa* | *Oryza sativa* |
| PpZEP | ppa002248m 100% | AT5G67030 67.61% | PT07G04430 71.43% | OS04G37619 62.37% |
| PpABA2 | ppa009814m 100% | AT1G52340 71.23% | PT01G02430 76.79% | OS03G59610 67.70% |
| PpNCED1 | ppa002804m 100% | AT3G14440 66.67% | PT01G39380 72.24% | OS03G44380 60.94% |
| PpNCED2 | ppa002314m 100% | AT1G30100 60.32% | PT11G08410 64.15% | OS03G44380 54.90% |
| PpNCED3 | ppa014647m 100% | AT3G24220 59.22% | PT03G17630 64.01% | OS03G44380 51.87% |
| PpNCED4 | ppa006109m 100% | AT4G19170 53.61% | PT19G09340 57.35% | No orthologue |
| PpAAO1 | ppa000263m 100% | AT2G27150 61.67% | PT09G15380 69.81% | OS07G18120 57.18% |
| PpCYP707A1 | ppa005059m 100% | AT4G19230 77.43% | PT04G23540 76.50% | OS02G47470 66.94% |
| PpCYP707A2 | ppa005020m 100% | AT2G29090 66.12% | PT09G03390 78.03% | OS02G47470 55.19% |
| PpCYP707A3 | ppa005226m 100% | AT3G19270 69.56% | PT04G14090 76.86% | OS09G28390 60.12% |
| PpCYP707A4 | ppa005234m 100% | AT3G19270 67.37% | PT14G02910 78.13% | OS09G28390 62.67% |

^1^Accession from Phytozome10.3: http://phytozome.jgi.doe.gov/pz/portal.html.

^2^Percentage identity calculated by pairwise alignments of protein sequences using DNAMAN6.0.
