# Supplementary material for: Expression of ABA Metabolism-Related Genes Suggests Similarities and Differences Between Seed Dormancy and Bud Dormancy of Peach (Prunus persica)
Source: Front Plant Sci. 2016 Jan 11;6:1248. doi: 10.3389/fpls.2015.01248 (PMC4707674; doi:10.3389/fpls.2015.01248)
Supplement: Supplementary file 3 [file Image1.PDF]

Expression of ABA metabolism-related genes suggests similarities and differences between seed dormancy and bud dormancy of peach (*Prunus persica*)

Dongling Wang[1,2 †], Zhenzhen Gao[1,2 †], Ling Li[1,2], Peiyong Du[1,2], Wei Xiao[1,2], Qiuping Tan[1,2], Xiude Chen[1,2], Dongsheng Gao[1,2]*

[1] State Key Laboratory of Crop Biology, Shandong Agricultural University, Taian, China

[2] College of Horticulture Science and Engineering, Shandong Agricultural University, Taian, China

[†] These authors contributed equally to this work.

* **Correspondence:** Dongsheng Gao, College of Horticulture Science and Engineering, Shandong Agricultural University, Tai'an, Shandong, China

dsgao@sdau.edu.cn

Supplementary Figures

Figure S1. Phylogenetic tree analysis of ABA metabolic genes from Arabidopsis and identified *Prunus Persica* (Pp-) orthologues. This was constructed by the neighbour-joining method with 1000 bootstrap replicates in the MEGA 6.0 software. The scale bar represents 0.2 substitutions per site

Figure S1

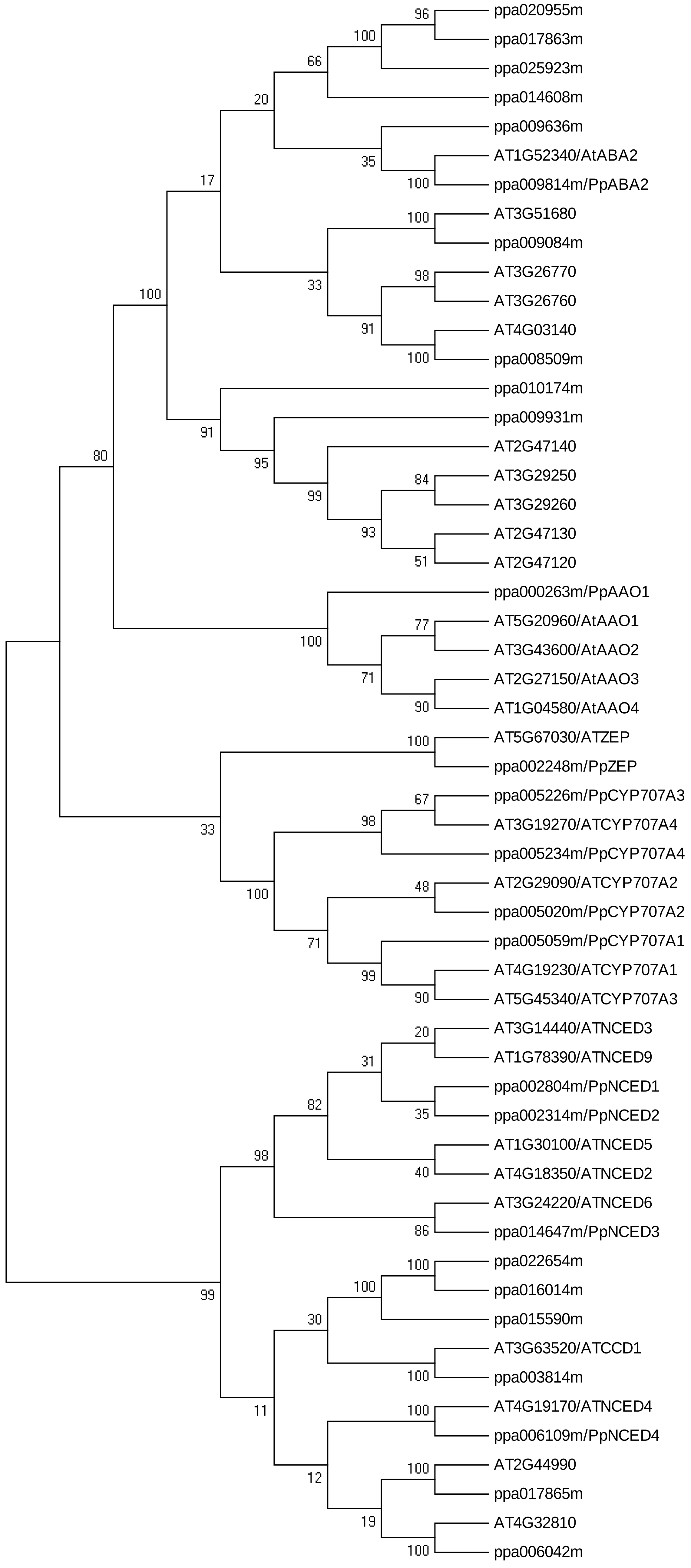
ppa020955m
ppa017863m
ppa025923m
ppa014608m
ppa009636m
AT1G52340/AtABA2
ppa009814m/PpABA2
AT3G51680
ppa009084m
AT3G26770
AT3G26760
AT4G03140
ppa008509m
ppa010174m
ppa009931m
AT2G47140
AT3G29250
AT3G29260
AT2G47130
AT2G47120
ppa000263m/PpAAO1
AT5G20960/AtAAO1
AT3G43600/AtAAO2
AT2G27150/AtAAO3
AT1G04580/AtAAO4
AT5G67030/ATZEP
ppa002248m/PpZEP
ppa005226m/PpCYP707A3
AT3G19270/ATCYP707A4
ppa005234m/PpCYP707A4
AT2G29090/ATCYP707A2
ppa005020m/PpCYP707A2
ppa005059m/PpCYP707A1
AT4G19230/ATCYP707A1
AT5G45340/ATCYP707A3
AT3G14440/ATNCED3
AT1G78390/ATNCED9
ppa002804m/PpNCED1
ppa002314m/PpNCED2
AT1G30100/ATNCED5
AT4G18350/ATNCED2
AT3G24220/ATNCED6
ppa014647m/PpNCED3
ppa022654m
ppa016014m
ppa015590m
AT3G63520/ATCCD1
ppa003814m
AT4G19170/ATNCED4
ppa006109m/PpNCED4
AT2G44990
ppa017865m
AT4G32810
ppa006042m
96
100
66
20
35
100
17
100
98
33
91
100
100
91
95
99
84
93
51
80
100
77
71
90
100
67
98
33
100
48
71
99
90
20
31
82
35
98
40
86
100
100
30
100
99
100
11
12
100
19
100
